# Organic compounds in water extracts of coal: links to Balkan endemic nephropathy

**DOI:** 10.1007/s10653-013-9515-1

**Published:** 2013-03-21

**Authors:** S. V. M. Maharaj, W. H. Orem, C. A. Tatu, H. E. Lerch, D. N. Szilagyi

**Affiliations:** 1 U.S. Geological Survey, Reston, VA 20192 USA; 2 Center for Research on Environmental Medicine, New Market, MD 21774 USA; 3 Department of Biology, University of Medicine and Pharmacy Timisoara, Timisoara, Romania; 4 Department of Pathology, County Hospital Timisoara, Timisoara, Romania

**Keywords:** BEN, Coal geochemistry, Environmental etiology, GC/MS, Groundwater simulation, Leaching experiments, Low-rank coal, Medical geology, Pliocene lignite hypothesis

## Abstract

The Pliocene lignite hypothesis is an environmental hypothesis that has been proposed to explain the etiology of Balkan endemic nephropathy (BEN). Aqueous leaching experiments were conducted on a variety of coal samples in order to simulate groundwater leaching of organic compounds, and to further test the role of the Pliocene lignite hypothesis in the etiology of BEN. Experiments were performed on lignite coal samples from endemic BEN areas in Romania and Serbia, and lignite and bituminous coals from nonendemic regions in Romania and the USA. Room temperature, hot water bath, and Soxhlet aqueous extraction experiments were conducted between 25 and 80 °C, and from 5 to 128 days in duration. A greater number of organic compounds and in higher concentrations were present in all three types of leaching experiments involving endemic area Pliocene lignite samples compared to all other coals examined. A BEN causing molecule or molecules may be among phenols, PAHs, benzenes, and/or lignin degradation compounds. The proposed transport pathway of the Pliocene lignite hypothesis for organic compound exposure from endemic area Pliocene lignite coals to well and spring drinking water, is likely. Aromatic compounds leached by groundwater from Pliocene lignite deposits in the vicinity of endemic BEN areas may play a role in the etiology of the disease. A better understanding of organic compounds leached by groundwater from Pliocene lignite deposits may potentially lead to the identification and implementation of effective strategies for the prevention of exposure to the causative agent(s) for BEN, and in turn, prevention of the disease.

## Introduction

Most of the world’s energy is provided by fossil fuels, and coal is the world’s most abundant fossil fuel with reserves substantially greater than those of oil and natural gas (Kavouridis and Koukouzas 2008). “Lignite” ranks in between peat and subbituminous in the sequential transformation from decomposed plant matter to anthracite (Whitehurst 1978). Balkan Peninsula countries rely heavily on lignite to meet their energy demands, as for example, it constitutes more than 85 % of the total coal reserves in Bulgaria (Siskov 1997), and generates over 60 % (Kavouridis and Koukouzas 2008) to more than 75 % (Iordanidis and Georgakopoulos 2003) of the total electric power output in Greece.

Coal use, however, has been associated with adverse human health effects (Wilson et al. 1980; Finkelman et al. 2002), and large health costs (Barbir and Veziroglu 1992). Coal quality parameters, for example, organic chemistry and leachability, have been identified as data needed for evaluating the potential human health impacts of coal (Finkelman and Gross 1999), as many adverse human health effects may be in part due to the mobilization of organic compounds (Swaine and Goodarzi 1995; Mumford et al. 1987, 1995; Mastrangelo et al. 1996; Finkelman et al. 1999). Organic compounds may be mobilized from coal into drinking water by groundwater, and by surface water, for example, during mining, and during transportation and storage prior to electric power plant utilization. Leached organic compounds from coal may also be hazardous to human health due to their potential to contribute to the formation of toxic compounds in the environment. Potential human health risks of organic compounds leached from coal include endocrine disruption, nephrotoxicity, and cancer (Gaitan et al. 1993; Bunnell et al. 2006; Finkelman 2007).

### Balkan endemic nephropathy

**Fig. 1:**
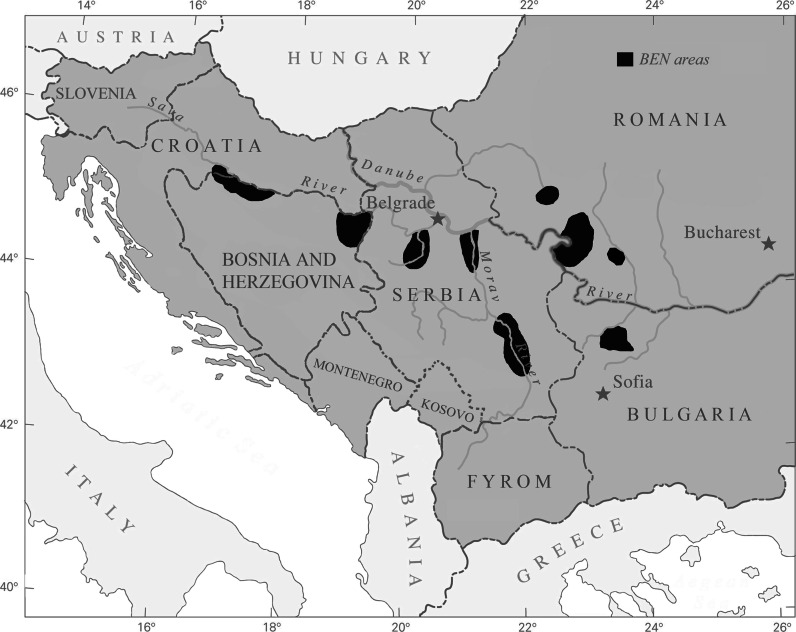
Map showing the distribution of Balkan endemic nephropathy regions (adapted after Tatu et al. 1998)

Balkan endemic nephropathy (BEN) is a chronic, tubulointerstitial renal disease often accompanied by urothelial cancer (Radovanovic and Krajinovic 1979; Vukelic et al. 1992), geographically confined to countries of the Balkan Peninsula with endemic locations in central and southeastern Serbia, southwestern Romania, northwestern Bulgaria, southeastern Croatia, and parts of Bosnia and possibly Kosovo (Fig. 1; Craciun and Rosculescu 1970; Feder et al. 1991; Ceovic et al. 1992). The disease occurs in rural villages, typically in alluvial valleys that contain a main drainage tributary of the Danube River. Prevalence of BEN in endemic areas is 2–10 % (Ceovic et al. 1992), lethality is nearly 100 % (Ceovic et al. 1992), and over 100,000 individuals may be at risk for developing the disease (Plestina 1992).

Hypotheses that have been proposed for the etiology of BEN include: lead contamination of wheat flour (Danilovic et al. 1957); heavy metals in water or foodstuffs (Gaon et al. 1962; Piscator et al. 1984; Wedeen 1991); autoimmunity (Craciun and Rosculescu 1970); aristolochic acid from *Aristolochia clematitis* seeds (Ivic 1970; Hranjec et al. 2005; Grollman and Jelakovic 2007); bacteria and viruses (Apostolov et al. 1975; Georgescu et al. 1976; Bozic et al. 1995); ochratoxin A, a fungal mycotoxin (Petkova-Bocharova et al. 1988); Pliocene lignites (Feder et al. 1991); selenium deficiency (Maksimovic 1991; Mihailovic et al. 1992); and genetic causes (Pavlovic et al. 1991, 2008; Toncheva et al. 1991; Toncheva and Dimitrov 1996). Strengths and limitations of hypotheses have been reviewed elsewhere (Plestina 1992; Stefanovic 1998; Tatu et al. 1998; Toncheva et al. 1998; Radovanovic 2002; Voice et al. 2006a, b; Long and Voice 2007; Gluhovschi et al. 2011a, b), but evidence from many studies (Batuman 1991; Morozov et al. 1991; Plestina 1992; Stefanovic 1998; Toncheva et al. 1998; Tatu et al. 1998; Radovanovic 2002; Stefanovic et al. 2006; Voice et al. 2006a) favors the involvement of one or more environmental factors, for example, exposure to Pliocene lignite coal deposits, in the etiology of the disease.

#### Pliocene lignite hypothesis

The Pliocene lignite hypothesis posits that BEN is caused by long-term exposure to low concentrations of aromatic hydrocarbons and other toxic organic compounds leaching into well and spring water via groundwater from low-rank coals, that is, Pliocene lignites, found in the vicinity of endemic settlements (Feder et al. 1991; Tatu et al. 1998; Orem et al. 1999). The rural population uses the well and spring water almost exclusively (Tatu et al. 2000a) for drinking, cooking, bathing, irrigation, and other purposes. The low concentration of the toxic organic compounds may account for the slow development (i.e., a 10–30-year subclinical incubation period) of the disease, and may possibly be linked to the high association of BEN with upper urinary tract carcinomas (Tatu et al. 1998; Orem et al. 1999), a form of cancer also with a much higher incidence in individuals from endemic versus nonendemic areas (Radovanovic and Krajinovic 1979; Cukuranovic et al. 1991; Petronic et al. 1991).

Limited information is available on the distribution of the coal deposits and the hydrology in the endemic areas (Radovanovic and Peric 1979; Feder et al. 1991, 2002; Tatu et al. 2000a). Geologic structure differs from one village to another (Craciun and Rosculescu 1970; Feder et al. 1991), but the bedrock adjacent to all endemic areas in the former Yugoslavia contain coal (Feder et al. 1991). In Romania and the former Yugoslavia, an 80–100-m column of Tertiary sediments would typically contain 10–15 Pliocene lignite layers (Feder et al. 2002). The mainly Pliocene age (1.8–5.3 million years old) coal layers in the endemic region are close to the surface, beginning 220–250 m in elevation at the western part of the coalfield, and decreasing to less than 100 m in elevation as they extend to the east/southeast/northeast. Therefore, the endemic villages may be located below the coal layers (located in the surrounding hills), or overlying the layers, and there are instances of wells penetrating through the coal layers, for example, in the endemic villages of Poroina, Romania and Vreoci, Serbia. In addition, in Romania, coal mines are in close proximity, that is, <1 km, to several endemic villages, for example, Erghevitza, Husnicioara, Livezile, Pietris, and Prunisor (Tatu et al. 2000a). In Bulgaria, extensive coal deposits occur north and south of the endemic region, and the endemic region is geologically defined by Pliocene sediments (Feder et al. 2002). Furthermore, in 2009, we discovered the presence of at least two types of coal in the Bulgarian endemic areas, one being a low-rank lignite, very similar to Romanian and Serbian endemic Pliocene lignites.

The well and spring water in endemic regions is largely supplied by aquifers with a relatively fast flow rate, for example, from rain. However, the underground transport pathway may be highly variable (Niagolova et al. 2005). The aquifers are shallow, as evidenced in part by the high nitrate levels (Orem et al. 2002; Niagolova et al. 2005), an indicator of surface contamination [that likely originate from fertilizers used by the villagers (Orem et al. 2002)]. In Romania, average depth-to-water of open shallow wells and springs in endemic villages is 0–10 m (Tatu et al. 2000a), and in Bulgaria it is <15 m (Niagolova et al. 2005). Given the relatively fast flow rate and the shallowness of the aquifers, it is unlikely that the well and spring water is supplied to a significant extent by, for example, fossil aquifers and paleowater.

#### Previous studies

Inorganic geochemical analyses of well and spring water samples from Romania have shown no significant differences between endemic and nonendemic regions (Orem et al. 2002, 2004), but for organic substances the situation is different. For example, a greater number of organic compounds (Goldberg et al. 1994) and in higher concentrations (Orem et al. 1999) were shown to be present in endemic versus nonendemic water samples, with well and spring water samples from endemic villages containing more aliphatic and aromatic compounds [many of which are potentially toxic (including, nitrogen-, oxygen-, and sulfur-containing heterocyclic compounds, aromatic amines, and phenols)], and in higher concentrations than water samples from nonendemic villages (Orem et al. 2002, 2004). Also, methanol extracts of lignite samples showed that endemic Pliocene coals possessed a complexity that was not matched by any nonendemic lignites, including a nonendemic Pliocene age lignite (Orem et al. 2002; Tatu et al. 2003), or other coals (Tatu et al. 2000a, b; Feder et al. 2002). In addition, human mesenchymal stem cells exposed to a water extract of an endemic area Pliocene lignite showed increased cellular proliferation and differentiation compared to the control (Suciu et al. 2005), and human kidney cells exposed to concentrated high-molecular-weight organic compounds from an endemic village water sample showed excess cell death or proliferation compared to controls (Bunnell et al. 2007).

However, a number of criticisms about the Pliocene lignite hypothesis, and many of the above previous studies have been raised (Pfohl-Leszkowicz et al. 2002; Voice et al. 2002, 2006a, b; Arlt et al. 2007; Gluhovschi et al. 2011a, b). For example, evaluations of the research that supports the Pliocene lignite hypothesis concluded that studies have not moved beyond a weak topographical association (Voice et al. 2006a, b). Demonstration of a cause-and-effect relationship was considered far away (Radovanovic 2002), in part because the source of the organic compounds in the well and spring water has been unconfirmed (Orem et al. 2004), and moreover, the exposure pathway has been deemed unlikely (Voice et al. 2006b).

Some limitations of the Pliocene lignite hypothesis have been addressed in the literature, for example, regarding the topographical association between the deposits and endemic BEN areas (Tatu et al. 1998; Finkelman et al. 2002). A comprehensive compilation of the possible limitations of the hypothesis, including whether other environmental agents may be more plausible as etiological factors than exposure to Pliocene lignites, for example, aristolochic acid from *A. clematitis* seeds (Ivic 1970; Hranjec et al. 2005; Grollman and Jelakovic 2007), with each limitation addressed in turn, is presented elsewhere (Maharaj 2013).

In addition, studies on Pliocene and other lignites from the Balkans, for example, Bulgaria (Stefanova et al. 2002), Greece (Siavalas et al. 2007), and Slovenia (Bechtel et al. 2003) have included organic geochemical analysis, but few studies have been conducted to identify organic compounds and their concentrations leached by water from coal experiments (McElmurry and Voice 2004). For example, experiments using coal samples (Koukouzas et al. 2010), including lignites (Baba and Kaya 2004; Koukouzas et al. 2009) have typically not included leaching experiments, and studies that have addressed the leaching of lignite samples (Orem et al. 1999; Wang et al. 2008; Izquierdo et al. 2011) have typically not included the identification of organic compounds.

#### This study

Therefore, we have conducted aqueous leaching experiments on a variety of coal samples, including Pliocene lignites from endemic BEN areas, in order to simulate groundwater leaching of organic compounds, and to further test the role of the Pliocene lignite hypothesis in the etiology of BEN. Herein, presented for the first time, is the identification and concentration of organic compounds leached by water from endemic (and nonendemic) area coal samples under various experimental conditions. Results provide direct evidence of the water-soluble, water-leachable, and water-extractable organic compounds, and therefore, evidence for also possibly linking the organic compounds present in the well and spring drinking water from endemic locations to the proximal Pliocene lignite coal deposits. Preliminary results only have been published (Orem et al. 2002, 2004; Finkelman et al. 2002; Tatu et al. 2003).

This study will add to the increasing body of knowledge in the re-emerging discipline known as medical geology (Finkelman et al. 2005; Selinus 2007), and may be used as part of an approach for potentially linking BEN in other areas, and/or other diseases, to organic compounds leached from coal by groundwater.

## Materials and methods

### Samples

The coal samples used in this study (Table 1) were Pliocene lignites from endemic BEN areas of Romania and Serbia, lignite and bituminous coals from nonendemic areas of Romania, and lignite and bituminous coals from nonendemic areas in the USA. Coal samples from Romania and Serbia were collected fresh from open pit and underground mines. Coal samples from the USA were obtained from the US Geological Survey (USGS) in Reston, VA, USA. All lignite coal samples available at the time of the experiments were used. Sample locations from Romania and Serbia were selected based on their endemic character, as determined from medical records obtained from the Drobeta Turnu Severin County Hospital in Romania, and in consultation with nephrophologists treating BEN patients in Serbia. Endemic regions are defined as areas with described, that is, known BEN cases (e.g., documented in medical records or the literature) and/or a family history of BEN. Nonendemic regions are defined as areas without any described BEN cases or a family history of the disease.

**Table 1:** Coal sample characteristics

Sample	Rank	Age	Locality	Region
Bpesyn	Lignite	Pliocene	Serbia	Endemic
Husnicioara	Lignite	Pliocene	Husnicioara, Romania	Endemic
Husnicioara pebbles	Lignite	Pliocene	Husnicioara, Romania	Endemic
Brad	Lignite	Miocene	Transylvania, Romania	Nonendemic
Haegel	Lignite	Paleocene	North Dakota, USA	Nonendemic
Doman	Bituminous	Carboniferous	Romania	Nonendemic
IRC #36	Bituminous	Carboniferous	Maryland, USA	Nonendemic

*IRC* internal reference coal. All sample grain sizes are <297 μm except for Husnicioara pebbles which are 5–10 mm in diameter

### Aqueous leaching experiments

Standard clean laboratory techniques for organic analyses were followed in cleaning all glassware and laboratory apparatus, and in sample preparation, analysis, and storage, including baking (450 °C) or rinsing of glassware with pesticide grade dichloromethane to remove any organic contaminants. Three types of experiments (Table 2) that simulate groundwater leaching of coals were performed: room temperature (25 °C), hot water bath (75 °C), and Soxhlet experiments (80 °C). Room temperature and hot water bath experiments were performed to approximate unheated and heated groundwater, respectively. As it would not have been feasible to leach organics from coal for decades in the laboratory (in order to mimic the subclinical exposure period of the disease), Soxhlet experiments were performed to accelerate leaching of organics to approximate groundwater leaching over long periods of time. However, due to the temperature condition used, hot water bath experiments would also likely accelerate leaching of organics, and therefore, this group of experiments may also be considered as approximating groundwater leaching over long periods of time, but to a lesser extent than Soxhlet experiments.

**Table 2:** Experimental runs and conditions

Sample #	Sample	Temp. (^o^C)	Time (days)
Rt-A-1	IRC #36	25	39
Rt-A-2	IRC #36	25	39
Rt-A-4	Husnicioara pebbles	25	39
Rt-C-4	Doman	25	97
Rt-C-5	Haegel	25	97
Rt-C-2	Brad	25	97
Rt-C-3	Brad	25	97
Rt-C-1	Bpesyn	25	97
Rt-B-1	IRC #36	25	128
Rt-B-2	IRC #36	25	128
Rt-B-3	Husnicioara	25	128
Rt-B-4	Husnicioara pebbles	25	128
Hb-A-1	IRC #36	75	5
Hb-A-2	IRC #36	75	5
Hb-A-6	Doman	75	5
Hb-A-5	Brad	75	5
Hb-A-3	Husnicioara	75	5
Hb-A-8	Husnicioara pebbles	75	5
Sx-F-1	IRC #36	80	5
Sx-F-2	IRC #36	80	5
Sx-F-7	Doman	80	5
Sx-F-8	Haegel	80	5
Sx-F-6	Brad	80	5
Sx-F-5	Bpesyn	80	5
Sx-F-3	Husnicioara	80	5
Sx-F-4	Husnicioara pebbles	80	5

*Rt* room temperature, *Hb* hot water bath, *Sx* Soxhlet

Ten grams of coal were used in each experiment. Samples were grounded to <297 μm grain size before leaching. One endemic area sample (i.e., Husnicioara) was also prepared as 5–10 mm diameter pebbles in order to compare possible differences in organic compound extractability as a function of grain size. Room temperature and hot water bath samples were placed in clean beakers with 100 and 250 ml milliQ water (i.e., 18 megaohms), respectively. Soxhlet coal samples were contained in Whatman cellulose extraction thimbles, and placed in Soxhlets with 250 ml milliQ water.

No attempt was made to mimic the elements or compounds present in natural groundwater in the water used for experiments, in order to ensure that results could be directly linked to experimental conditions, as possible side interactions could have occurred due to the presence of introduced solutes. In addition, distilled, deionized, and/or demineralized water has been commonly used in the laboratory in coal leaching experiments (Praharaj et al. 2002; Wang et al. 2008; Chakraborty and Mukherjee 2009; Ruhl et al. 2010; Yuan et al. 2010), including with lignite samples (Tatu et al. 1998; Orem et al. 1999; McElmurry and Voice 2004; Izquierdo et al. 2011), and to study organic compounds (Koopmans et al. 1997; Kruger et al. 2012) or metals (Prokop et al. 2003) from other geologic material. Furthermore, laboratory experiments that simulated groundwater transport have often not mimicked aquifer ion composition (Bales et al. 1997; Prommer et al. 2008; Jung et al. 2009; Wright et al. 2010; Zhang et al. 2010), particularly when the species of the metals are not known (Christensen and Christensen 1999), as is the case here. Finally, it is unlikely that the inorganic parameters of the water have a significant effect on the extractability of the organic compounds.

Duplicate experiments were performed under each experimental condition (Table 2) for quality assurance. Samples were held at maximum temperature for the entire duration of the experiments. After the aqueous extraction period was completed, the water from the coal extraction was filtered (while still hot, for hot water bath and Soxhlet runs) using clean (baked at 450 °C) Whatman GF/C glass microfiber filters (1 μm pore size). Pesticide grade dichloromethane (30 ml) was added to the filtered water extract for stabilization. Samples were liquid–liquid extracted with 3 sequential volumes of 30 ml pesticide grade dichloromethane to isolate the organic compounds in the filtered aqueous extract. The combined extract (120 ml) was concentrated by rotoevaporation, followed by evaporation under nitrogen. Samples were brought up in 50–2,000 μl pesticide grade dichloromethane, and 1–2 μl each was used for analysis by gas chromatography (GC), and gas chromatography/mass spectrometry (GC/MS).

### Analytical methods

Samples were analyzed using a Perkin Elmer (Boston, MA, USA) 8500 GC with a Flame Ionization Detector (FID) and a Hewlett Packard (Agilent Technologies, Palo Alto, CA, USA) 6890 Series GC System with a 5973 Mass Selective Detector equipped with an Agilent 7683 Series Injector. Samples were first analyzed by GC–FID in order to determine whether separation by liquid chromatography (LC) was needed prior to GC/MS analysis. A 30 m × 250 μm × 0.25 μm J&W DB-5 column (95 % dimethyl, 5 % diphenyl polysiloxane) was used for GC–FID analysis using the following conditions: 1.0 μl splitless injection; injector temperature of 150 °C; detector temperature of 325 °C; temperature program of 70–325 °C at 4 °C/min with a final hold at 325 °C for 20 min; and 13 psig column pressure. LC columns were packed with silicic acid (BIOSIL A, 100–200 mesh) and aluminum oxide (Neutral Alumina AG7, 100–200 mesh).

GC/MS was used for structural identification and quantification of the organic compounds. A 30 m × 250 μm × 0.25 μm HP-5MS column (95 % dimethyl, 5 % diphenyl polysiloxane) was used for GC/MS analysis, with the following conditions: 1–2 μl splitless injection; a constant flow of 1.0 ml/min; solvent delay of 6.0 min; injector temperature of 150 °C; interface at 300 °C; temperature program of 70–300 °C at 4 °C/min with a final hold at 300 °C for 15 min; and a mass scan from 50 to 550 Da (Dalton).

Laboratory, GC, and GC/MS blanks were used for quality assurance. Laboratory blanks consisted of milliQ water extracted with pesticide grade dichloromethane, subjected to the same processing conditions as samples. GC and GC/MS blanks were run prior to each analysis. Organic compounds in samples were identified by comparison of mass spectral features to reference libraries of mass spectral data [National Institute of Standards and Technology (NIST98) and Wiley 7] included in the GC/MS system, and interpreted visually, or by comparison to standard compounds. Compounds identified at a match quality of ≥90 % against the mass spectral databases are reliably identified. Match qualities between 89 and 50 % may be considered tentatively identified, and those <50 % should be considered unreliable, and the compound should be considered unknown or unidentified. Average ion composition was calculated for each peak, and peak deconvolution was performed on all peaks.

External aliphatic and aromatic standards, injected under the same conditions as samples, were used for quantification. Due to the impracticality of preparing standards with hundreds of compounds, aliphatic standards consisted of 32 compounds (C8 through C40) at 1 and 4 ppm concentration, and aromatic standards consisted of 23 major compounds, prepared in part from EPA 610 (and contained, for example, acenaphthene, acenaphthylene, anthracene, benz(a)anthracene, benzo(a)pyrene, benzo(b)fluoranthene, benzo(g,h,i)perylene, benzo(k)fluoranthene, chrysene, dibenz(a,h)anthracene, fluoranthene, fluorene, indeno(1,2,3-cd)pyrene, naphthalene, phenanthrene, pyrene) at 1 and 10 ppm concentration. To quantitate compounds in samples, a subset of 3 standard compounds with known retention times were chosen for each sample. Compounds in samples were quantified using standards with retention times that best corresponded to the retention time ranges (i.e., ≤25, 26–44, and ≥45 min) of the sample compounds. As the majority of sample compounds were not quantified with the actual standard for that compound, the majority of sample compound concentration results are semi-quantitative. Inferential statistics were not employed because group sizes were too small for results, whether they would have shown statistical significant or no statistical significance, to have been meaningful.

## Results

Laboratory blank concentrations of organic compounds were very low, and show that contamination associated with laboratory methods, for example, extraction and rotoevaporation, was negligible. All GC and GC/MS blank runs showed no peaks discernable above background, indicating no sample or standard carryover.

Table 3 lists the total number of organic compounds present in samples [at abundances proportional to ≥25,000 counts (~0.01 ng/μl) for a 1/50-μl injection], and percentages of organic compounds identified at match qualities of ≥90, 89–50, and <50 %. Some compounds listed as a match quality of 89–50 % may have been a ≥90 % match quality, as the GC/MS scans began at 50 m/z (atomic mass to charge ratio). Tables 4 and 5 list the percentages and concentrations, respectively, of aliphatic and aromatic compound groups with a match quality of ≥90 %. Experiments in Tables 2, 3, 4, 5 are grouped by run conditions, and within each experimental subgroup by sample origin, that is, nonendemic followed by endemic area coals.

**Table 3:** Number and percentages of organic compounds in samples

Sample #	Region and rank	No. of compounds present	Percentage identified match ≥90 %	Percentage identified match 89–50 %	Percentage identified match <50 %
Rt-A-1	Nonendemic bituminous	29	41	24	35
Rt-A-2	Nonendemic bituminous	11	46	27	27
Rt-A-4	Endemic lignite	47	23	53	23
Rt-C-4	Nonendemic bituminous	5	80	0	20
Rt-C-5	Nonendemic lignite	28	46	39	14
Rt-C-2	Nonendemic lignite	9	56	33	11
Rt-C-3	Nonendemic lignite	12	17	50	33
Rt-C-1	Endemic lignite	31	16	45	39
Rt-B-1	Nonendemic bituminous	6	50	50	0
Rt-B-2	Nonendemic bituminous	5	40	40	20
Rt-B-3	Endemic lignite	34	12	44	44
Rt-B-4	Endemic lignite	19	16	53	32
Hb-A-1	Nonendemic bituminous	84	43	36	21
Hb-A-2	Nonendemic bituminous	76	32	32	37
Hb-A-6	Nonendemic bituminous	38	58	32	11
Hb-A-5	Nonendemic lignite	28	32	36	32
Hb-A-3	Endemic lignite	117	19	42	39
Hb-A-8	Endemic lignite	172	19	32	49
Sx-F-1	Nonendemic bituminous	95	13	47	40
Sx-F-2	Nonendemic bituminous	102	15	42	43
Sx-F-7	Nonendemic bituminous	112	21	46	33
Sx-F-8	Nonendemic lignite	392	5	17	78
Sx-F-6	Nonendemic lignite	193	8	35	57
Sx-F-5	Endemic lignite	278^a^	6	34	60
Sx-F-3	Endemic lignite	352^a^	7	18	75
Sx-F-4	Endemic lignite	301^a^	6	23	71

*Rt* room temperature, *Hb* hot water bath, *Sx* Soxhlet

^a^Contains 200–400 more compounds

**Table 4:** Percentages of aliphatic and aromatic compounds (≥90 % match) in samples

Sample #	Aliphatic compounds			Aromatic compounds										
	Saturated	Unsaturated	Functional derivatives	Phthalate esters				Phenols			PAHs	Other		
				Dibutyl phthalate	Diethyl phthalate	Diethyl-hexyl phthalate	Other phthalates	N-, P- phenols	Hydroxy-, methoxy-phenols	Other (esters, etc.)	Phenanthrene, fluoranthene and derivatives	Benzenes (hydroxy-, methoxy-, etc.)	Heterocyclic with N, O, or S	Lignin degradation (vanillin group)
Rt-A-1	8	8	42	8	8	−	8	−	8	−	−	−	8	−
Rt-A-2	−	20	−	20	20	20	−	−	−	−	−	−	20	−
Rt-A-4	36	9	9	9	9	18	−	−	−	−	−	−	9	−
Rt-C-4	−	−	−	25	25	−	50	−	−	−	−	−	−	−
Rt-C-5	78	−	−	−	8	8	−	−	8	−	−	−	−	−
Rt-C-2	−	−	20	20	20	20	20	−	−	−	−	−	−	−
Rt-C-3	−	−	−	−	50	50	−	−	−	−	−	−	−	−
Rt-C-1	40	−	20	−	−	20	−	−	−	−	−	−	20	−
Rt-B-1	−	−	−	−	33	−	33	−	−	33	−	−	−	−
Rt-B-2	−	−	−	−	50	50	−	−	−	−	−	−	−	−
Rt-B-3	−	−	−	−	25	25	−	−	50	−	−	−	−	−
Rt-B-4	−	−	−	−	33	33	−	−	−	−	−	33	−	−
Hb-A-1	39	3	11	3	3	6	11	8	6	−	−	3	6	−
Hb-A-2	46	−	4	4	4	13	13	4	4	−	−	4	4	−
Hb-A-6	64	−	−	5	5	5	5	5	−	5	−	−	5	−
Hb-A-5	11	−	−	−	11	−	11	−	22	−	−	33	−	11
Hb-A-3	14	−	23	−	5	−	−	−	18	9	−	27	−	5
Hb-A-8	22	−	13	−	−	3	−	−	22	6	−	22	3	9
Sx-F-1	−	−	25	−	8	8	−	−	8	−	8	42	−	−
Sx-F-2	−	−	7	−	−	13	−	−	7	7	7	53	7	−
Sx-F-7	−	−	43	−	4	4	−	−	13	4	4	23	4	−
Sx-F-8	−	−	5	−	−	5	−	−	19	19	10	38	−	5
Sx-F-6	−	−	27	−	−	7	7	−	13	−	13	33	−	−
Sx-F-5	−	−	6	−	−	−	−	−	47	−	18	29	−	−
Sx-F-3	−	−	12	−	−	−	−	−	32	4	8	40	−	4
Sx-F-4	−	−	18	−	−	6	−	−	24	−	6	35	−	12

−, No compounds at the match quality specified, that is, ≥90 %

**Table 5:** Concentration (ng/g) of aliphatic and aromatic compounds (≥90 % match) in samples

Sample #	Aliphatic compounds			Aromatic compounds										
	Saturated	Unsaturated	Functional derivatives	Phthalate esters				Phenols			PAHs	Other		
				Dibutyl phthalate	Diethyl phthalate	Diethyl-hexyl phthalate	Other phthalates	N-, P- phenols	Hydroxy-, methoxy-phenols	Other (esters, etc.)	Phenanthrene, fluoranthene and derivatives	Benzenes (hydroxy-, methoxy-, etc.)	Heterocyclic with N, O, or S	Lignin degradation (vanillin group)
Rt-A-1	5.74	3.26	11.40	77.49	22.74	−	10.41	−	0.11	−	−	−	10.06	−
Rt-A-2	−	1.47	−	45.71	4.76	70.33	−	−	−	−	−	−	11.28	−
Rt-A-4	5.19	1.96	5.53	23.06	16.25	49.61	−	−	−	−	−	−	13.95	−
Rt-C-4	−	−	−	2.24	14.22	−	5,247.81	−	−	−	−	−	−	−
Rt-C-5	26.71	−	−	−	7.39	33.11	−	−	1.42	−	−	−	−	−
Rt-C-2	−	−	1.26	1.86	7.87	35.02	3.44	−	−	−	−	−	−	−
Rt-C-3	−	−	−	−	9.67	87.41	−	−	−	−	−	−	−	−
Rt-C-1	4.82	−	1.64	−	−	31.90	−	−	−	−	−	−	3.67	−
Rt-B-1	−	−	−	−	279.37	−	2.36	−	−	729.86	−	−	−	−
Rt-B-2	−	−	−	−	183.05	295.37	−	−	−	−	−	−	−	−
Rt-B-3	−	−	−	−	34.99	2,381.91	−	−	35.56	−	−	−	−	−
Rt-B-4	−	−	−	−	21.72	165.95	−	−	−	−	−	2.22	−	−
Hb-A-1	46.91	17.05	27.85	29.21	32.16	78.60	800.88	117.84	29.03	−	−	3.19	71.99	−
Hb-A-2	39.34	−	0.55	23.24	109.78	34.76	706.72	4.45	3.72	−	−	3.96	26.41	−
Hb-A-6	70.72	−	−	15.20	10.57	19.19	485.62	4.44	−	3.51	−	−	18.97	−
Hb-A-5	129.48	−	−	−	16.10	−	490.31	−	13.23	−	−	125.08	−	31.61
Hb-A-3	7.21	−	24.62	−	261.26	−	−	−	682.28	2,639.87	−	614.82	−	2,703.36
Hb-A-8	23.18	−	14.31	−	−	1,465.77	−	−	1,711.98	22.56	−	603.66	54.90	9,593.10
Sx-F-1	−	−	180.54	−	4.10	4.86	−	−	1.80	−	13.01	347.88	−	−
Sx-F-2	−	−	58.67	−	−	8.42	−	−	20.14	1.13	15.87	310.90	4.30	−
Sx-F-7	−	−	1,894.46	−	4.12	21.34	−	−	120.75	4.38	3.73	174.35	147.76	−
Sx-F-8	−	−	583.54	−	−	444.76	−	−	3,259.91	200.69	174.50	24,318.31	−	2,821.79
Sx-F-6	−	−	1,097.44	−	−	26.04	79.57	−	1,634.62	−	121.08	14,405.35	−	−
Sx-F-5	−	−	673.64	−	−	−	−	−	33,690.48	−	1,003.33	55,988.01	−	−
Sx-F-3	−	−	2,085.83	−	−	−	−	−	1,456.96	16.04	91.68	13,633.61	−	3,179.94
Sx-F-4	−	−	378.31	−	−	840.51	−	−	3,768.64	−	321.83	88,650.51	−	14,983.86

−, No compounds at the match quality specified, that is, ≥90 %

**Fig. 2:**
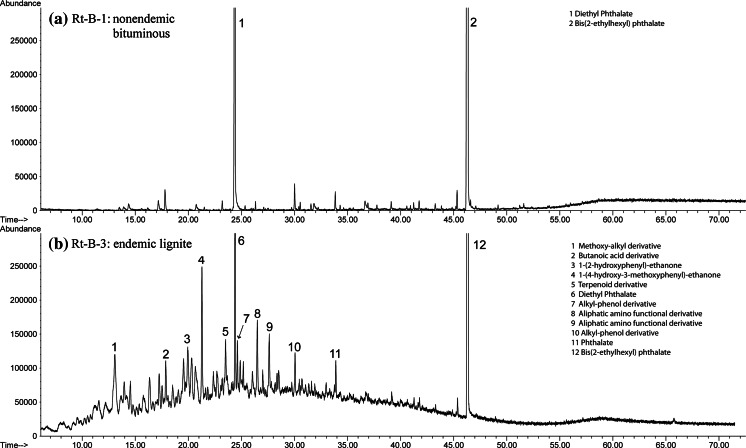
GC–MS chromatographs of room temperature experiments held at 25 °C for 128 days comparing bituminous coal and endemic area lignite samples. **a** Rt-B-1, a bituminous coal (i.e., IRC #36), and **b** Rt-B-3, an endemic area lignite sample (i.e., Husnicioara) showing more organic compounds and in higher concentrations than in **a**

**Fig. 3:**
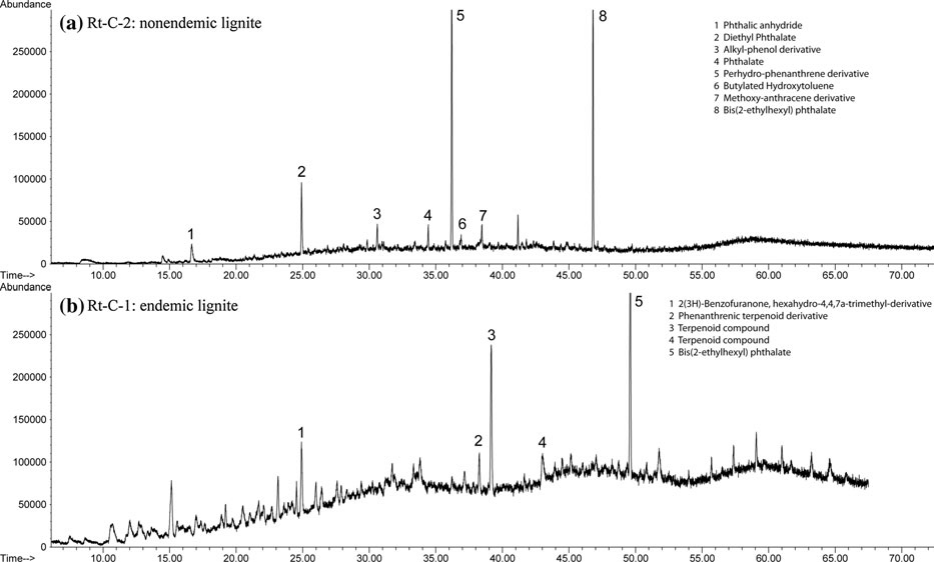
GC–MS chromatographs of room temperature experiments held at 25 °C for 97 days comparing nonendemic and endemic area lignite samples. **a** Rt-C-2, a nonendemic area lignite sample (i.e., Brad), and **b** Rt-C-1, an endemic area lignite sample (i.e., Bpesyn) showing more organic compounds and in higher concentrations than in **a**

Duplicate aqueous extraction experiments show consistency in aliphatic and aromatic compound concentrations (Table 5). Room temperature aqueous extraction experiments using endemic area Pliocene lignite samples produced many more organic compounds and in greater concentrations than bituminous coals run under identical conditions (Fig. 2). Room temperature experiments using an endemic area Pliocene lignite sample also produced many more organic compounds and in greater concentrations than nonendemic area lignites from Romania and the USA run under identical conditions (Fig. 3).

**Fig. 4:**
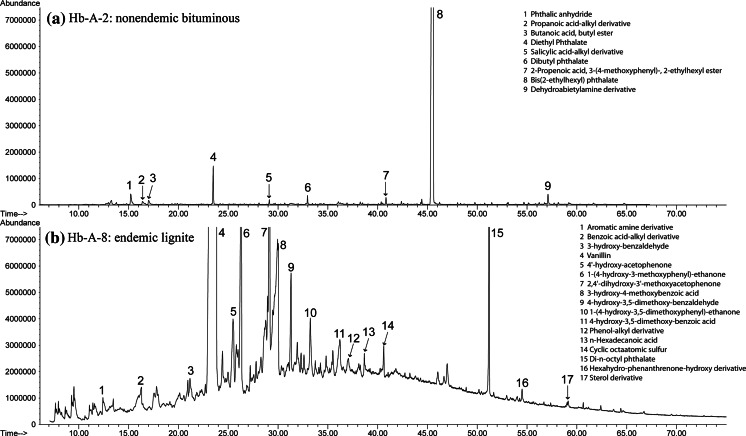
GC–MS chromatographs of hot water bath experiments held at 75 °C for 5 days comparing bituminous coal and endemic area lignite samples. **a** Hb-A-2, a bituminous coal (i.e., IRC #36), and **b** Hb-A-8, an endemic area lignite sample (i.e., Husnicioara pebbles) showing more organic compounds and in higher concentrations than in **a**

**Fig. 5:**
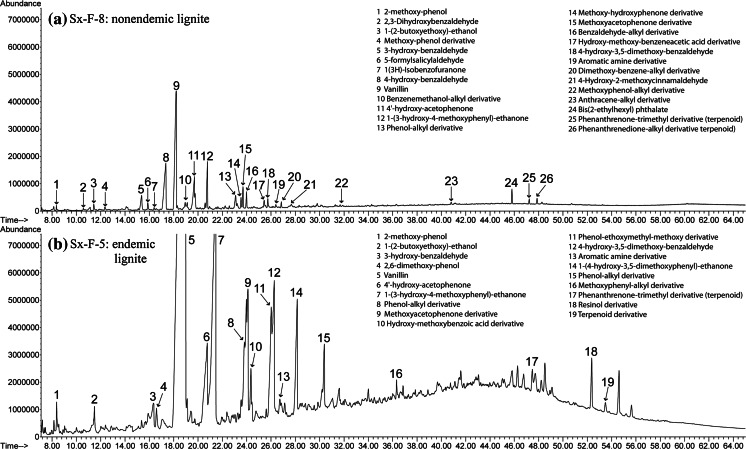
GC–MS chromatographs of Soxhlet experiments held at 80 °C for 5 days comparing nonendemic and endemic area lignite samples. **a** Sx-F-8, a nonendemic area lignite sample (i.e., Haegel), and **b** Sx-F-5, an endemic area lignite sample (i.e., Bpesyn) showing more organic compounds and in higher concentrations than in **a**

Similar results were obtained for hot water bath experiments. Aqueous extracts of endemic area Pliocene lignites contained more organic compounds and in higher concentrations than nonendemic area samples (Fig. 4). Hot water bath experiments yielded a higher concentration of saturated aliphatic compounds, phthalate esters, phenols, benzenes, heterocycles, and lignin degradation compounds than room temperature experiments (Table 5). Aqueous Soxhlet extraction experiments produced much more complex GC/MS chromatograms than either room temperature or hot water bath extraction experiments, and also showed more and higher concentrations of organic compounds extracted from endemic area Pliocene lignites compared to all other coals (Fig. 5). Soxhlet experiments also yielded a higher concentration of functional derivative aliphatics, hydroxy-, methoxy-phenols, PAHs, hydroxy-, methoxy-benzenes, and lignin degradation compounds than room temperature or hot water bath experiments (Table 5).

Pebble-sized Husnicioara samples run under higher temperature experimental conditions (i.e., 75° and 80 °C) produced a higher concentration of aromatic compounds than corresponding finer grained (<297 μm) Husnicioara samples, particularly for the lignin degradation subgroup.

Many samples contained phthalate esters, ubiquitous environmental contaminants of anthropogenic origin. As laboratory blank concentrations of organic compounds were very low, the phthalate esters represent contamination of the coal prior to processing in the laboratory.

## Discussion

Greater numbers and higher concentrations of organic compounds were present in aqueous leachates of endemic area Pliocene lignites compared to the other coals examined. Though some organic compound information from extracts of bituminous coals have been presented before (Zhao et al. 2000; Tatu et al. 2000a, b; 2003; Orem et al. 2002; Feder et al. 2002), and we expected these higher ranking coals to have lower numbers and concentrations of organic compounds than lignites, components extracted from coal vary depending on, for example, the solvent, temperature, and time used; therefore, their inclusion in this study was necessary in order to directly compare coal sample extract yields under the experimental conditions used.

Results of leaching experiments using lignite samples may be influenced by different grain sizes because of differences in surface area (Izquierdo et al. 2011). The higher concentration of aromatic compounds in the higher temperature (i.e., 75° and 80 °C) pebble-sized Husnicioara sample experiments versus corresponding finer grained (<297 μm) sample experiments suggest leaching from coal sample surface exteriors, as the coarser grained samples possessed higher ratios of total exposed exterior surface area:interior grain surface area.

Similar concentrations of saturated and unsaturated aliphatic compounds and their functional derivatives were found in aqueous leachates of endemic area Pliocene lignites and the other coals examined, suggesting that these compounds do not play an etiological role in BEN. Many aromatic compounds, for example, PAHs and aromatic amines, are known or suspected carcinogens, or have been linked to urinary tract cancer and tubulointerstitial nephropathies (Tatu et al. 1998). A BEN causative agent or agents may be among the numerous aromatic compound classes identified in the extracts (phenols, PAHs, benzenes), as for example, in the experiments designed to gauge exposure to groundwater leaching of organic compounds over long periods of time (i.e., hot water bath and Soxhlet), hydroxy-, methoxy-phenols and hydroxy-, methoxy-benzenes occur in much higher concentrations in extracts of endemic area Pliocene lignites compared to the other coals and conditions examined.

As the majority of samples contain ≥50 % of their compounds identified with match qualities of 89–50 or <50 %, a specific BEN causative molecule or molecules may also be among these compounds, or among the hundreds of compounds present in lower concentrations than addressed in this study. The relatively low concentrations of compounds (in the ng to μg/g range) present in samples, however, may provide a plausible explanation for the extended time required for kidney failure and upper urinary tract cancer to develop in BEN patients.

Many organic compounds in endemic area Pliocene lignite coal extracts were also found in water supply samples (Orem et al. 2004) of endemic (but not nonendemic) regions in Romania, including cycloalkanes/alkenes, steranic structures, monoaromatic and polyaromatic terpanes, polycyclic aromatic hydrocarbons, and aromatic amines, suggesting a connection and link to the source of the compounds found in the water. In addition, phenolic compounds and aromatic derivatives present in the water supply samples were attributed to either anthropogenic pollution or a geologic source (Orem et al. 2004). However, the higher concentrations of hydroxy-, methoxy-phenols, PAHs and derivatives, and hydroxy-, methoxy-benzenes present in the higher temperature extracts of endemic area Pliocene lignites (compared to extracts of other coals), show that the organics detected in the endemic water supply samples are more consistent with a geologic source, that is, Pliocene lignites.

Thus, this study shows that many aromatic compounds present in endemic area Pliocene lignite coals are water-soluble (at least in part, as particles are ≤1 μm), water-leachable, and water-extractable. The proposed exposure pathway of the Pliocene lignite hypothesis (i.e., organic compounds in endemic area Pliocene lignites → leached by groundwater → organic compounds in well and spring drinking water) is therefore likely, and indicates (with the presented results) that the source of some of the organic compounds in endemic area water supply samples is the proximal Pliocene lignite coal deposits.

However, exposure to organic compounds leached from Pliocene lignite coal deposits into well and spring drinking water in endemic BEN areas alone cannot explain the etiology of the disease, as for example, not all individuals residing in endemic villages develop BEN. Genetic susceptibility is also involved in the emergence of the disease. For example, the gene-environment interaction is translated into unusual xenobiotic substance metabolism (controlled by cytochrome *P*450 and other enzymes) that increases the risk to develop BEN only in those individuals that bear certain gene variants that code for the detoxification enzymes (Atanasova et al. 2005).

### Future work

Additional research on this subject is warranted for an improved understanding of the role of the Pliocene lignite hypothesis in the etiology of BEN. Additional work should address the identification and concentration of compounds with match qualities of 89–50 %, and of compounds with lower concentrations than investigated here. Also, a compound-by-compound assessment for known or suspected causal links to tubulointerstitial nephropathies and urinary tract cancers appear needed, as well as a compound-by-compound comparison of endemic and nonendemic area extract yields from this study to those of water supply samples. Future experiments should be conducted on a greater number of samples, using longer time durations, and with water that better mimics the natural inorganic groundwater composition. Coal samples for future experiments should include Pliocene lignites from neighboring countries with no known cases of BEN, for example, Greece, Slovenia, and Turkey, in order to further narrow the comparison of coals to similar rank and flora.

## Conclusions

This study supports the role of the Pliocene lignite hypothesis as a factor in the etiology of Balkan endemic nephropathy. Water-soluble, water-leachable, and water-extractable aromatic compounds with functional groups with potential toxicity have been demonstrated to be leached from endemic area Pliocene lignite coal samples under a number of experimental conditions. A BEN causing molecule or molecules may be among phenols, PAHs, benzenes, and/or lignin degradation compounds, as these compounds occurred in greater concentrations in extracts of endemic area Pliocene lignite sample experiments that best mimicked long-term compound exposure, compared to all other coals and conditions examined. In addition, some of these same compounds have been identified in endemic (but not nonendemic) area water supply samples, and therefore, indicate a link to the proximal Pliocene lignite coal deposits. The concentrations of organic compounds are low, in the ng to μg/g range, and may account for the long time required for development of the disease. The proposed transport pathway of the Pliocene lignite hypothesis for organic compound exposure from endemic Pliocene lignite coals to well and spring drinking water, is therefore, likely.
